# Early surgical treatment of closed reduction and internal fixation for a 30-day old intertrochanteric fracture with hemiplegia after acute stroke: A case report

**DOI:** 10.1097/MD.0000000000034098

**Published:** 2023-06-23

**Authors:** Zeng-Li Zhang, Xu-Song Li, Wei-Qiang Zhao, Jie-Feng Huang, Ya-Hong Zhu

**Affiliations:** a Department of Orthopaedics and Traumatology, Songyang Hospital of Traditional Chinese Medicine, Songyang, China; b Department of Orthopaedics and Traumatology, Zhongshan Hospital of Traditional Chinese Medicine, Zhongshan, China; c The First Clinical College, Zhejiang Chinese Medical University, Hangzhou, China; d Department of Orthopaedics and Traumatology, The First Affiliated Hospital of Zhejiang Chinese Medical University, Hangzhou, China; e Emergency Center, The First Affiliated Hospital of Zhejiang Chinese Medical University, Hangzhou, China.

**Keywords:** acute, intertrochanteric fracture, old, stroke, surgical treatment

## Abstract

Currently, there are no clear guidelines to determine whether and when to perform surgical hip repair in patients with acute stroke and hip fracture.

**Patient concerns:**

In this case report, we report a case of 75-year-old woman admitted with left hip pain and limited mobility for 1 month.

**Diagnoses:**

Patient had a history of acute cerebral infarction 42 days ago, and diagnosed with a left intertrochanteric fracture at another hospital 30 days ago.

**Intervention:**

Patient was treated with closed reduction and internal fixation with proximal femoral nail anti-rotation.

**Outcomes:**

At 2-year follow-up, the patient’s basic function was restored. The fracture healed well, and the Harris hip score was 75.

**Lessons:**

Without consistent guidelines, individualized treatment strategies including surgical methods and timing of surgery should be made to weigh the risks and benefits for patients with acute stroke and intertrochanteric fractures.

## 1. Introduction

Hip fractures are a significant post-stroke complication.^[1,2]^ The incidence of hip fracture in stroke patients with hemiplegia is 2 to 4 times higher than that of healthy people.^[3–7]^ Paradoxically, hip fractures in elderly also increase stroke risk,^[8]^ and needing early surgical treatment to reduce morbidity and mortality.^[9]^ Prior studies have shown that hemiplegia after acute stroke combined with hip fracture leads to worse neurological recovery, prolonged hospitalization period, increased complications, decreased patient prognosis, and increased 30-day and one-year mortalities.^[3,4]^

The surgical treatment goals of intertrochanteric fractures were fracture stabilization, early patient mobilization, and restoring to previous level of independence and function.^[10]^ The mainstream treatment is internal fixation (including intramedullary and extramedullary), and hip arthroplasty has also been reported.^[11–13]^ However, there is no accepted surgical procedure for old intertrochanteric fractures. At the same time, acute cerebral infarction is a relative contraindication to hip reconstructive surgery, therefore, most of these patients cannot undergo surgical treatment.^[4]^ In general, elective surgery after acute stroke is best delayed by 2 weeks, preferably 6 weeks. However, there are no clear guidelines to determine whether and when to perform surgical hip repair in patients with acute stroke and hip fracture.

We report a case of an old femoral intertrochanteric fracture with hemiplegia after acute stroke, treated with closed reduction and internal fixation (CRIF) with proximal femoral nail anti-rotation (PFNA).

## 2. Case presentation

Informed consent was obtained from the patient for publication of this case report details.

**Figure 1.**
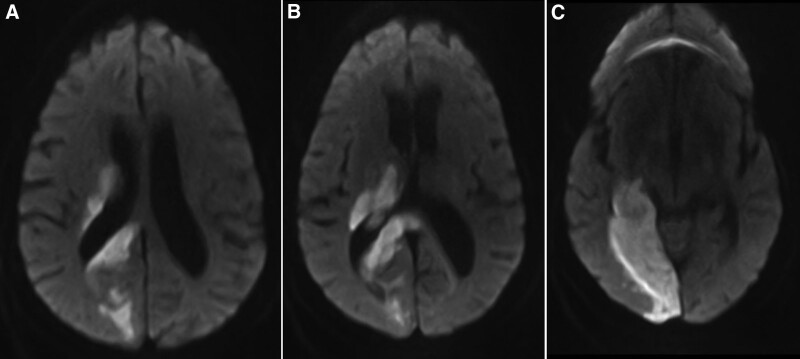
The brain MRI on the day of onset. Diffusion weighted imaging (DWI) scan showed hyperintensity shadow in the right paraventricular, corpus callosum and temporo-occipital lobe.

A 75-year-old woman was admitted with left hip pain and limited mobility for 1 month. She had a history of acute cerebral infarction 42 days ago (Fig. 1), and tripped during rehabilitation exercise 30 days ago. She was diagnosed with a left intertrochanteric fracture at another hospital, but the operation was postponed due to acute cerebral infarction.

**Figure 2.**
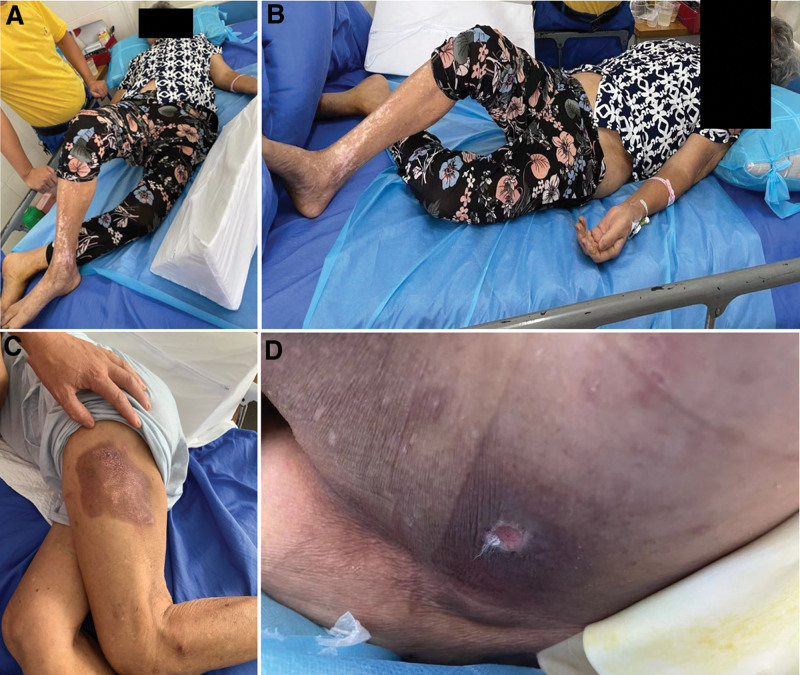
Left hip deformity and skin condition preoperative. A and B: The patient’s left lower limb showed obvious adduction and external rotation deformity. C: The skin of the left hip appeared drug eruption due to external use of drugs. D: A third-degree pressure sores on the sacral tail skin.

The patient’s left lower limb showed obvious adduction and external rotation deformity (Fig. 2A and B). The skin of the left hip appeared skin rash due to external use of Chinese herbs (Fig. 2C), and there was a third-degree pressure sores on the sacral tail skin (Fig. 2D). The muscle strength was grade I of left lower limb, and grade III of left upper limb. Neurological function scores were evaluated preoperative, the National Institutes of Health stroke scale^[14]^ was 7, and the activity daily living was heavily dependent and the Barthel index^14,15]^ was 30.

**Figure 3.**
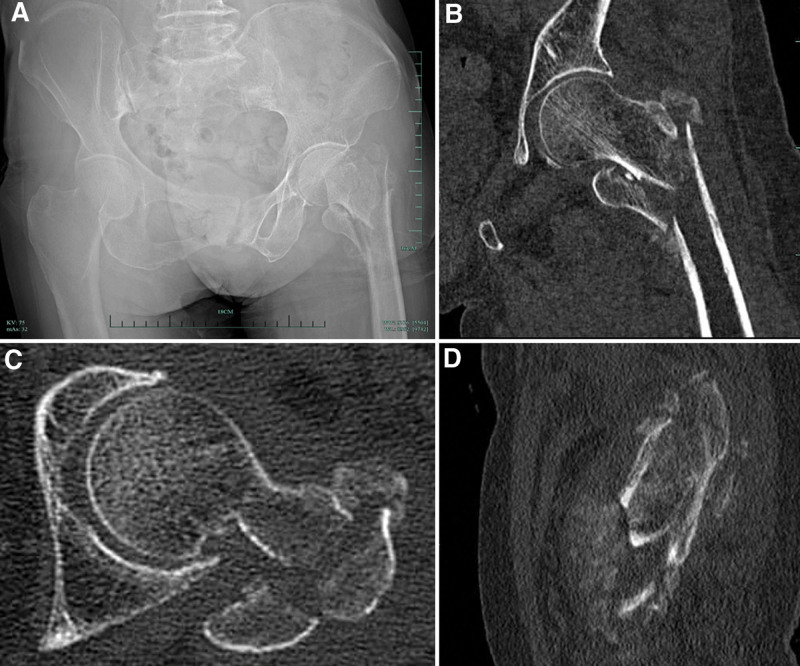
Preoperative imaging findings of the patient. Radiographs (A), coronal (B), transverse (C), and sagittal (D). CT scans identified a left intertrochanteric fracture accompanied by greater and lesser trochanteric fractures with varus deformity, and callus growth at the fracture end. CT = computed tomography.

Radiographs and computed tomography identified a type I, group IV intertrochanteric fracture according to Evans classification (Fig. 3). The Dual-energy X-ray absorptiometry of the right hip was examined, and the T-score was −3.9.

**Figure 4.**
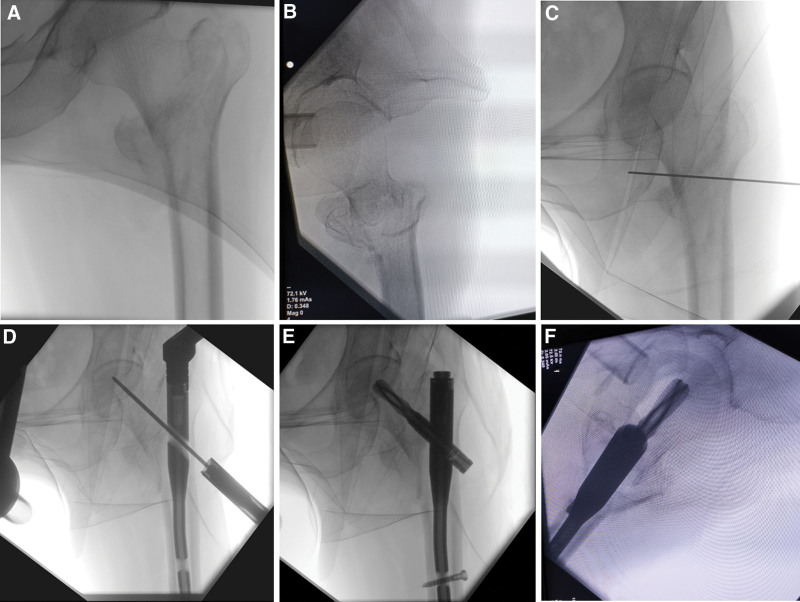
Intraoperative imaging findings of the patient. A and B: After closed reduction, C-arm fluoroscopy was performed under traction showed Intertrochanteric fracture of the left hip had basically reached anatomical reduction. C: Preoperative body surface positioning under traction bed. D–F: PFNA was placed during the operation. PFNA = proximal femoral nail anti-rotation.

After successful epidural anesthesia, the patient was placed in the supine position. The partially bone union was simply re-fractured using closed reduction manipulation, and the left lower limb was maintained traction and rotated inward, then c-arm fluoroscopy was performed. When the fracture had basically reached anatomical reduction, the patient underwent CRIF with percutaneous PFNA on traction bed (Fig. 4).

Cefuroxime (1.5 g intravenously twice a day) was given 24 hours perioperative to prevent infection. Low molecular weight heparin (1500 μL subcutaneous injection once per day) was given to prevent deep vein thrombosis, and was bridged with aspirin (0.1 g orally, once a day) 5 weeks after surgery. Atorvastatin (20 mg orally, once a day) was used to prevent recurrence of stroke. Anti-osteoporosis therapy was also used.

The patient was encouraged to move early on the bed with endurable pain. Postoperative rehabilitation treatment was continued twice a day, including physical therapy, aerobic training and physical agents therapy. Specific rehabilitation training measures need to be evaluated and adjusted according to the actual situation of the patient. Table 1 lists the details of rehabilitation methods. Acupuncture was also performed once a day.

**Table 1:** The details of the patient’s rehabilitation methods.

Item	
Physical therapy	
Use bare hands in bed	Muscle strength training, focusing on hip flexors and quadriceps
	Range of motion training, mainly active and passive range of motion of the hip and knee
	MOTOmed training of limb in bed
	Routine maintenance of the healthy side and left upper limb
Bedside sitting or standing* exercises	Bobath shake hands and roll over exercise
	Help off the bed turn to sit up, sit on the bed
	Sitting balance training, from sitting to standing
	Standing balance training, and the use of rehabilitation aids
Gait training*	Shift of weight, stride and step
Aerobic Training	Diaphragmatic breathing exercises
	Resistance training of upper and lower limbs assisted with elastic bandage
	Assisted hand or power bike training
Physical agents therapy	Neuromuscular electrical stimulation
	Infrared ray therapy†
	Ultrashort wave therapy†
	Transcranial magnetic stimulation

* When the patient’s left lower limb muscle strength reached grade III or above, standing training and gait training was performed.

† Use within 2 weeks after surgery.

**Figure 5.**
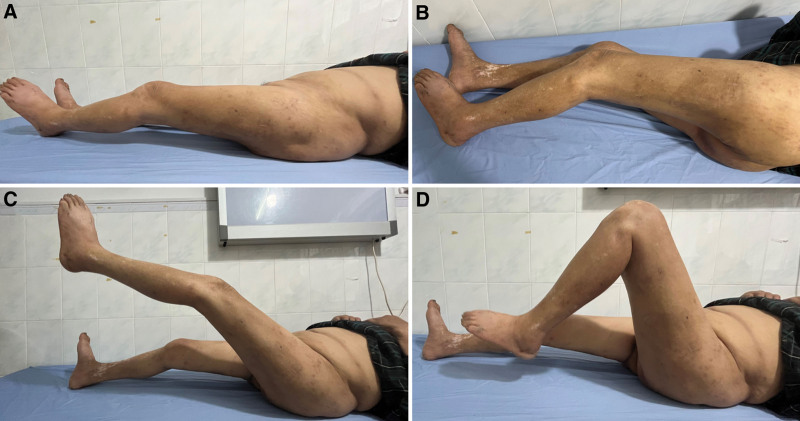
The incision healed well and basic function was restored. The incision healed well and basic function was restored.

**Figure 6.**
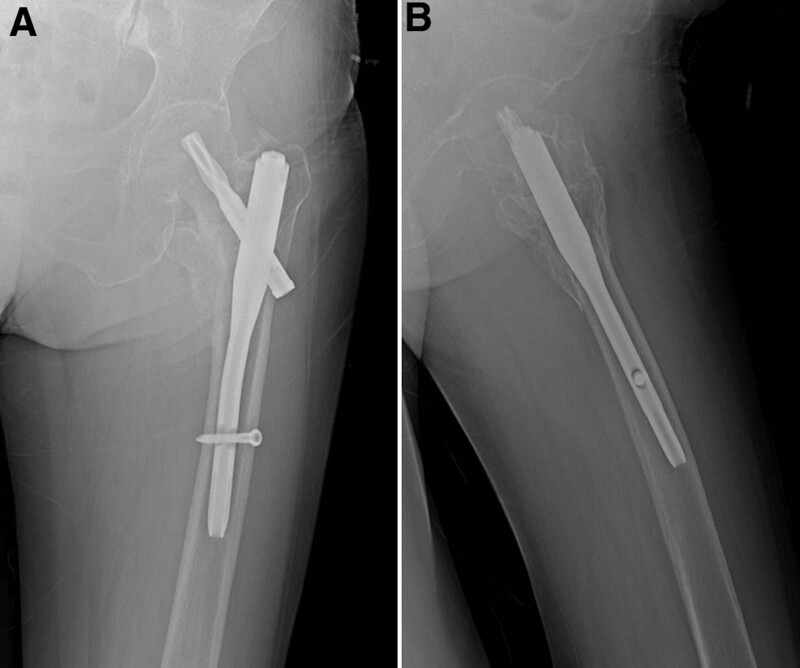
Radiographs of the left hip showed good fracture healing at 2-year follow-up. At 2-year follow-up, anteroposterior (A) and lateral (B) radiographs of the left hip showed good fracture healing.

The pressure ulcer healed 2 weeks after operation. At 12 weeks postoperatively, the fracture healed radiographically and the patient was able to stand on support or with the aid of a walker. At 2-year follow-up, the muscle strength was grade IV of left lower limb, and grade V of left upper limb. The National Institutes of Health stroke scale was 0, and the activity daily living was independent and the Barthel index was 80. The fracture healed well, and the Harris hip score was 75 (Figs. 5 and 6).

## 3. Discussion

Although acute cerebral infarction is a relative contraindication to surgery, a small sample study (11 cases) in which the patients and family members requested and insisted on hip reconstructive surgery has reported that surgical treatment can improve the prognosis of patients with acute cerebral infarction.^[4]^ However, the study did not address the timing of surgery or the surgical treatment method of hip reconstruction. A case of intertrochanteric fracture and acute stroke, focusing on individual factors to reduce postoperative complications and recurrent stroke rate by a multidisciplinary team, treated with PFNA has been reported.^[16]^ In our case, the 30-day intertrochanteric fracture had local callus growth at the fracture end on images, which may due to brain injury.^[17]^ Meanwhile, acute post-stroke hemiplegia, pressure sores, and drug eruption of the hip make treatment more difficult.

We prepared 2 surgical options. According to the healing process of the 30-day intertrochanteric fracture, the fracture ends have been connected by cartilage callus, and hard callus gradually appeared. At this point, closed reduction was still possible. Intraoperative fluoroscopy after closed reduction also confirmed our judgment. If closed reduction fails, hip arthroplasty would be chosen.

CRIF with PFNA has 3 advantages. First, it corrected the hip deformity and relieved the pain. Second, compared with hip arthroplasty, the operation is less traumatic and conducive to postoperative rehabilitation. Third, revision hip arthroplasty is still available after failed fixation.^[18]^ Hemiplegia, pressure sores, and drug eruption of the hip may increase the risk of dislocation and infection after hip arthroplasty, therefore, only considered as an alternative to the failure of closed reduction. At the same time, total hip arthroplasty for old intertrochanteric fracture is more difficult procedure than for osteonecrosis or osteoarthritis.

In conclusion, without consistent guidelines, individualized treatment strategies including surgical methods and timing of surgery should be made to weigh the risks and benefits for patients with acute stroke and intertrochanteric fractures.

## Author contributions

**Conceptualization:** Zeng-Li Zhang, Jie-Feng Huang, Ya-Hong Zhu.

**Data curation:** Zeng-Li Zhang, Xu-Song Li, Wei-Qiang Zhao.

**Formal analysis:** Xu-Song Li.

**Investigation:** Xu-Song Li, Wei-Qiang Zhao, Ya-Hong Zhu.

**Validation:** Jie-Feng Huang.

**Writing – original draft:** Zeng-Li Zhang.

**Writing – review & editing:** Jie-Feng Huang, Ya-Hong Zhu.
